# A three-dimensional computed tomography study to determine the ideal method for fluoroscopically-guided bone marrow aspiration from the iliac crest

**DOI:** 10.17305/bjbms.2020.4744

**Published:** 2021-06

**Authors:** Ryan S. D’Souza, Langping Li, Shuai Leng, Christine Hunt, Luke Law, Casey Muir, Jason Eldrige, Mohamad Bydon, Meng Chi, Shane Shapiro, William D. Mauck, Wenchun Qu

**Affiliations:** 1 Department of Anesthesiology and Perioperative Medicine, Mayo Clinic, Rochester, Minnesota, USA; 2 Department of Physical Medicine and Rehabilitation, Mayo Clinic, Rochester, Minnesota, USA; 3 Department of Anesthesiology, Ruijin Hospital, Luwan Branch, Shanghai Jiao Tong University School of Medicine, Shanghai, China; 4 Department of Pain Medicine, Mayo Clinic, Rochester, Minnesota, USA; 5 Department of Neurosurgery, Mayo Clinic, Rochester, Minnesota, USA

**Keywords:** Mesenchymal stem cells, bone marrow aspirate, regenerative medicine

## Abstract

Bone marrow aspiration (BMA) through the iliac crest is potentially unsafe due to the vicinity of neurovascular structures in the greater sciatic notch. Our objective was to investigate the safety of a recently described BMA technique, specifically a trajectory from the posterior superior iliac spine (PSIS) to the anterior inferior iliac spine (AIIS). We conducted a chart review of 260 patients, analyzing three-dimensional (3D) reconstructed computed tomography (CT) images of the pelvis and sacrum to validate that this new approach offers a wide safety margin from the greater sciatic notch. Analysis of 3D CT scans demonstrated that the PSIS to AIIS trajectory never crossed the greater sciatic notch. The trajectory was noted to be at least one cm away from the greater sciatic notch in all measurements. The new trajectory entered the PSIS at 25.29 ± 4.34° (left side) and 24.93 ± 4.15° (right side) cephalad from the transverse plane, and 24.58 ± 4.99° (left side) and 24.56 ± 4.67° (right side) lateral from the midsagittal plane. The area of bone marrow encountered with the new approach was approximately 22.5 cm^2^. Utilizing the same CT scans, the trajectory from the traditional approach crossed the greater sciatic notch in all scans, highlighting the potential for violating the greater sciatic notch boundary, and damaging important neurovascular structures. Statistically, significant sex-related differences were identified in needle trajectory angles for both approaches. We conclude that based on this 3D CT study, a trajectory from the PSIS to the AIIS for BMA may offer a wide safety margin from the greater sciatic notch.

## INTRODUCTION

Mesenchymal stem cells (MSCs) are an important cornerstone in cell-based transplantation and regenerative medicine therapy [1,2]. Their immune-modulatory and trophic effects may ameliorate the degenerative process, augment tissue healing in the case of injury, provide pain relief, and support functional recovery with the goal of delaying surgery and improving quality of life [3-5]. Bone marrow aspiration (BMA) is a common procedure utilized to harvest MSCs.

Two major considerations are essential when obtaining high-quality bone marrow aspirate. Safety is the highest priority. While BMA is generally considered a safe percutaneous procedure, concern for severe adverse events exists, notably nerve injuries and hemorrhage [5,6]. Secondly, avoidance of blood dilution is paramount to obtain a sufficient concentration of MSCs. Additionally, a high volume of bone marrow is often required and is obtained at structures with the most cancellous bone [4].

**FIGURE 1:**
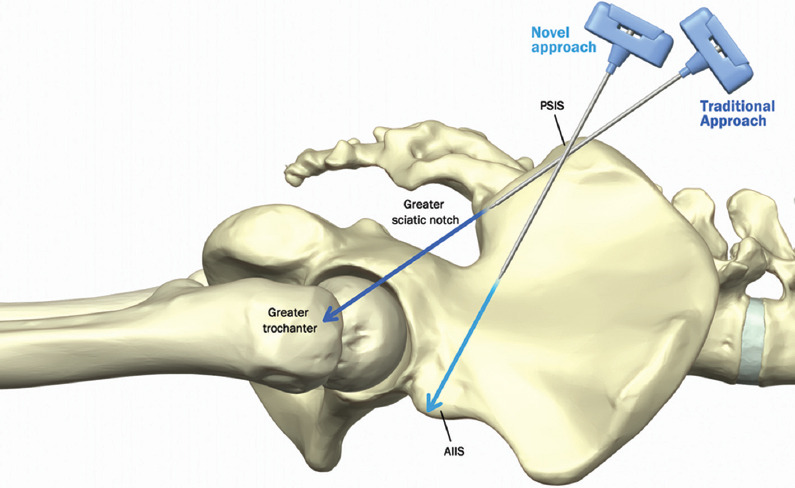
Traditional and new approach of bone marrow aspiration. Traditional approach: Needle is angled 20-45° caudal from the transverse plane and 30-45° lateral from the parasagittal plane, with the advancement of the needle taking place between the inner and outer tables of the ilium through the posterior superior iliac spine (PSIS) to the greater trochanter. New approach describes a proposed needle trajectory from PSIS to anterior inferior iliac spine.

Currently, there is no standardized technique for BMA. Although the ilium is the preferred source of bone marrow for aspiration of MSCs, the approach, trajectory angle, and depth of the needle path are highly variable. While no consensus needle aspiration techniques have been described through the iliac crest, some authors suggest angling the needle approximately 20-45° caudal from the transverse plane and 30-45° lateral from the parasagittal plane, with the advancement of the needle taking place between the inner and outer tables of the ilium through the posterior superior iliac spine (PSIS) towards the greater trochanter (Figure 1) [4,7-11]. However, this practice may be inefficient due to limited bone marrow aspirate concentration (BMAC) yield necessitating multiple aspiration attempts [1,12-14], and more importantly, potentially hazardous because of the vicinity to the greater sciatic notch, through which run important neurovascular structures. Specific nerves at risk for damage include the superior gluteal nerve manifesting with a disabling Trendelenburg gait, and the sciatic nerve manifesting with leg weakness and paresthesias [4,15]. Vascular injury to the superior gluteal artery and vein may lead to a hematoma, pain, and compartment syndrome [3,5,16,17]. These concerns warrant investigation of a better BMA approach that offers optimal BMAC yield and minimizes the risk of such adverse events.

**FIGURE 2:**
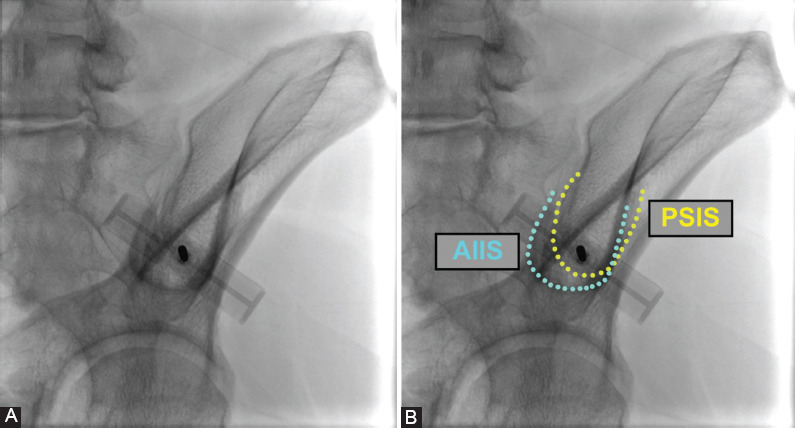
(A) Novel fluoroscopic-guided approach to bone marrow aspiration. Fluoroscopic image displays the “double teardrop” view with the posterior superior iliac spine (PSIS) projecting over the ASIS with the cortical margins aligned. The needle is advanced from the lower margin of the teardrop from the PSIS toward the anterior inferior iliac spine (AIIS). (B) Boundaries are outlined and labeled for both the AIIS and PSIS in the “double teardrop” view.

We propose an approach with the needle trajectory from the PSIS aimed toward the anterior inferior iliac spine (AIIS). This technique utilizes a fluoroscopic-guided “obturator-outlet” view, or “teardrop-merging” view, as the PSIS and AIIS should be superimposed radiographically with the needle advancing coaxially (Figure 2). When inserted to a maximum depth of 6 cm, this trajectory may ensure safe passage between the tables of the ilium, while avoiding the greater sciatic notch. A similar BMA approach was described by Hirahara et al. [18], although there were notable differences from our proposed approach. Hirahara et al. defined the trajectory as an angle between the vector from the most medial aspect of the PSIS through the ilium and a vector going through the midline of the pelvis, while our study employed a trajectory from the PSIS to the AIIS [18]. In addition, Hirahara et al. described an ultrasonography-based technique, while we proposed a fluoroscopically-guided needle injection utilizing the obturator-outlet view [18].

The objective of our theoretical model study was to utilize three-dimensional (3D) reconstructed computed tomography (CT) images of the pelvis and sacrum to validate that this recently described approach (needle trajectory from PSIS to AIIS) offers a wide safety margin that averts injury to the neurovascular structures within the greater sciatic notch.

## MATERIALS AND METHODS

### Study overview

This was a retrospective study of 260 Caucasian patients who had undergone 3D reconstructed CT images of the pelvis and sacrum between April 11, 2015, and November 4, 2016. These images were analyzed to characterize an ideal trajectory for needle insertion during BMA, specifically the trajectory between the PSIS and AIIS and the relationship of this trajectory to the greater sciatic notch and other structures of the pelvis. It should be noted that patients did not have an actual BMA procedure; they had CT scans of the pelvis and sacrum which were subsequently utilized to identify the feasibility and safety of theoretical needle trajectories for BMA.

### Setting

We performed this study at a major tertiary referral center (Mayo Clinic Hospital, Rochester, MN). This review was approved by the Mayo Clinic Institutional Review Board (IRB# 16-008082).

### Selection of participants

Adults (age 18-80) who were patients at the Mayo Clinic and underwent CT imaging of the sacrum and pelvis as part of routine clinical care were included in the study. Patients who displayed significantly distorted anatomy due to congenital or acquired abnormalities, including but not limited to spina bifida, bony dysplasia, spinal fusion, and severe ankylosing spondylosis, were excluded from the study.

### Outcome measures

**FIGURE 3:**
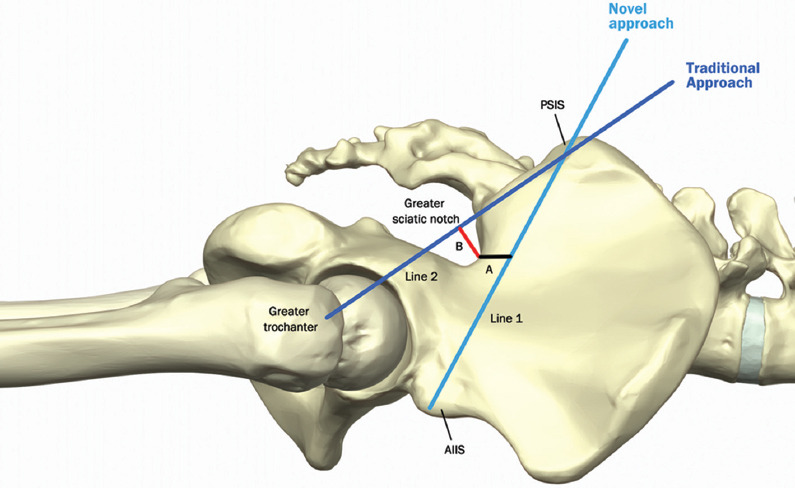
Aspiration approach and safety margin from greater sciatic notch. Line 1 connects posterior superior iliac spine (PSIS) to anterior inferior iliac spine (new Bone marrow aspiration [BMA] approach), and A refers to distance between line 1 and the greater sciatic notch. Line 2 connects PSIS to greater trochanter (traditional BMA approach), and B refers to distance between line 2 and the greater sciatic notch.

**FIGURE 4:**
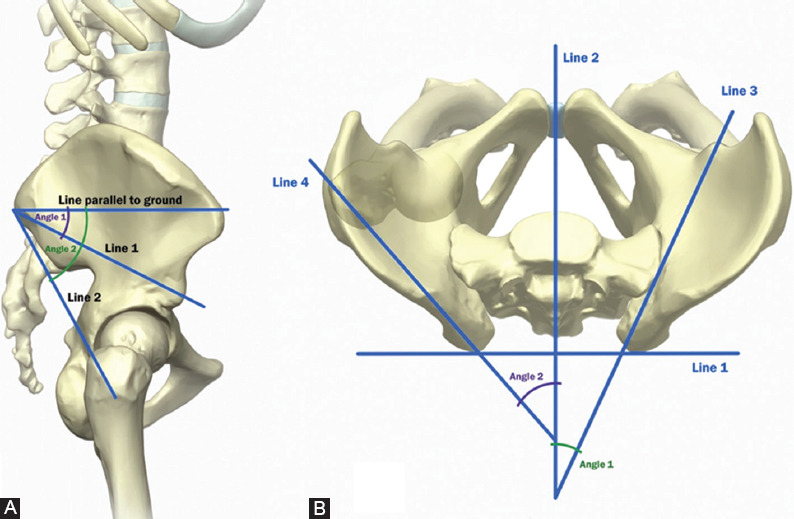
(A) Anatomic diagram displaying cephalad angulation. Line 1 connects posterior superior iliac spine (PSIS) to anterior inferior iliac spine (AIIS) (new Bone marrow aspiration [BMA] approach) and line 2 connects PSIS to the greater trochanter (traditional BMA approach). Angle 1 indicates cephalad angulation for the new BMA approach, and angle 2 indicates cephalad angulation for the traditional BMA approach. (B) Anatomic diagram displaying contralateral obliquity. Line 1 connects the PSIS from both sides, line 2 is a perpendicular line to line 1 and is centered at midline, line 3 connects PSIS to AIIS (new BMA approach), and line 4 connects PSIS to greater trochanter (traditional BMA approach). This forms an angle at the intersection between line 2 and 3, referred to as angle of contralateral obliquity. Angle 1 indicates contralateral obliquity fwor the new BMA approach, and angle 2 indicates contralateral obliquity for the traditional BMA approach.

Baseline demographic characteristics were assessed, including age, sex, and ethnicity. 3D reconstructed CT pelvis and sacrum from 260 patients were reviewed and pertinent landmarks were identified, including the PSIS, AIIS, and greater trochanter. As a measure of safety margin of the new BMA approach (needle from PSIS to AIIS), we measured the distance between the greater sciatic notch and a needle trajectory from the PSIS to AIIS (Figure 3); specifically, the distance was measured as a perpendicular line from the needle trajectory to the closest point on the greater sciatic notch. For the traditional approach (needle from PSIS to greater trochanter), we measured the distance between the greater sciatic notch and a needle trajectory from the PSIS to the greater trochanter (Figure 3); specifically, the distance was measured as a perpendicular line from the needle trajectory to the farthest point on the greater sciatic notch boundary. The reason for choosing a trajectory from the PSIS to the greater trochanter for the traditional approach stems from studies and protocols demonstrating that this approach crosses the posterior-most sector of the iliac crest, which has the greatest thickness of cancellous iliac bone and maybe most appropriate for optimizing bone marrow aspirate volume [4,9-11]. The degree of cephalad angulation and contralateral obliquity were also assessed for both approaches, reflecting the degree of the needle trajectory required to keep the needle between the two cortical bone plates of the iliac crest in the sagittal plane and axial plane, respectively (Figure 4). Finally, we measured the area of the entire iliac crest cross section encountered with the entire needle trajectory from the PSIS to AIIS; since a second aspiration attempt is frequently performed, we also reported the area of the entire iliac crest cross section encountered by a needle trajectory that is 30° cephalad to the new trajectory.

### Statistical analysis

The mean and standard deviations (SD) for all continuous dependent variables were calculated. Student’s *t*-test was used to make comparisons between continuous outcomes. Comparisons were made between right-sided and left-sided measurements within each study arm. Comparisons were also made for all outcomes between the two cohorts. A separate subgroup analysis was also performed in both study arms to compare all measurements between male and female sex to determine sex-based differences. All analyses were performed using SPSS (IBM SPSS Statistics for Windows, Version 21.0; Armonk, NY: IBM Corp.). Statistical significance was accepted at a *p* ≤ 0.05.

## RESULTS

Our final sample consisted of 182 of patients. Seventy-eight patients (30% of total) were excluded from our cohort due to unclear radiographic images or distorted anatomy. All patients in our cohort were Caucasian. Eighty-one patients were males (44.5%) and 101 were females (55.5%). The average age was 60 ± 18 years.

All outcomes of interest are displayed in Table 1. Utilizing the new BMA approach, the shortest distance between the greater sciatic notch and a needle trajectory between the PSIS and AIIS was 1.91 ± 0.35 cm on the left side and 1.89 ± 0.31 cm on the right side. In all the CT scans, none of the needle ­trajectories utilizing the new BMA approach crossed the greater sciatic notch and all were noted to be at least 1 cm above the greater sciatic notch in all measurements. The degree of cephalad angulation was 25.29° ± 4.34° on the left side and 24.93° ± 4.15° on the right side, and the degree of contralateral obliquity was 24.58° ± 4.99° on the left side and 24.56° ± 4.67° on the right side.

**TABLE 1:**
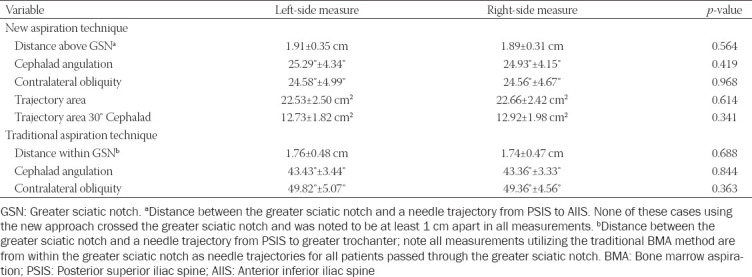
Chart with primary and secondary outcomes

The area of bone marrow encountered with the new trajectory from PSIS to AIIS was 22.53 ± 2.50 cm^2^ on the left side and 22.66 ± 2.42 cm^2^ on the right side. The area encountered by the needle trajectory 30° cephalad to the new trajectory was 12.73 ± 1.82 cm^2^ on the left side and 12.92 ± 1.98 cm^2^ on the right side.

Utilizing the traditional approach, the needle trajectory always crossed the greater sciatic notch in all included CT scans. The distance between the greater sciatic notch boundary and a needle trajectory from the PSIS to the greater trochanter was 1.76 ± 0.48 cm on the left side and 1.74 ± 0.47 cm on the right side (*p* < 0.001 and *p* < 0.001, respectively, when compared to measurements from new technique). The degree of cephalad angulation was 43.43° ± 3.44° on the left side and 43.36° ± 3.33° on the right side (*p* < 0.001 and *p* < 0.001, respectively, when compared to measurements from new technique), and the degree of contralateral obliquity was 49.82° ± 5.07° on the left side and 49.36° ± 4.56° on the right side (*p* < 0.001 and *p* < 0.001, respectively, when compared to measurements from new technique).

There were no statistically significant differences between all left-sided and right-sided measurements in both study arms. Subgroup analysis comparing measurements between male and female sex are displayed in Supplemental Table 1. Notably, statistically significant sex-related differences were present across most variables of needle trajectory angle in both study arms.

## DISCUSSION

BMA techniques merit scrutiny as MSCs are increasingly utilized in regenerative therapy for musculoskeletal disease. Although various approaches have been investigated to acquire bone marrow [1,19,20], limited evidence exists on the safety and quality of these techniques. Utilizing fluoroscopic guidance, a newly described approach to BMA was described as a “teardrop merging” view that allows for easy and safe passage of a needle trajectory from the PSIS to AIIS [21-23]. Our study utilized 3D reconstructed CT images of the pelvis and sacrum to characterize and validate the safety and area of bone encountered during BMA from this new technique. Based on the results of our study, a needle trajectory from the PSIS to the AIIS for BMA is preferable due to a wider safety margin from the greater sciatic notch and higher area of cancellous bone encountered by the trocar.

Regarding measures of safety, the new approach was superior to the traditional approach as the former was noted to never cross the greater sciatic notch in all CT scans. An average distance of 1.9 cm between the needle trajectory and the greater sciatic notch was noted, providing a very wide safety margin for error. In fact, a distance of at least 1 cm between the needle trajectory and the greater sciatic notch was noted in all measurements.

The safety of this new approach from the PSIS to the AIIS has also been described in the orthopedic literature in patients undergoing iliac screw placement for sacral fractures, high-grade spondylolisthesis, lumbosacral fracture dislocation, and instability from spondylodiscitis and fusion failure [23,24] A study by Vilela et al. [24] demonstrated that placement of a long iliac screw can be achieved using the obturator outlet view which provides accurate information of the screw position and integrity of the acetabulum, sciatic notch, and iliac buttress cortices; in a series of 38 separate iliac screw placements, there were no neurovascular injuries, acetabulum or sciatic notch violations, and loosened or ­fracture screws [24].

Utilizing the traditional approach, the potential danger of violating the boundary of the greater sciatic notch is highlighted by our observation that the needle trajectory crossed the greater sciatic notch in all included CT scans. Moreover, it is even more concerning that the needle trajectory in the traditional approach is deep within the greater sciatic notch, averaging a depth of 1.76 ± 0.48 cm on the left side and 1.74 ± 0.47 cm on the right side. This underscores the importance of limiting trocar advancement to a superficial depth, as deeper advancement will likely violate the boundary of the greater sciatic notch structures. This is consistent with several prior studies demonstrating risk for sciatic nerve and gluteal vessel damage when the trocar was inserted deeper than 6 cm into the posterior iliac crest [4].

The disparity of needle trajectory angle between the two approaches is further exemplified by the difference in cephalad angulation and contralateral obliquity. The degree of cephalad angulation was approximately 25° for the new approach, while it was approximately 43° for the traditional approach, reflecting a prominent difference of 18°. The divergence is even wider with measures of contralateral obliquity, measuring at approximately 25° in the new approach, and approximately 50° in the traditional approach. The trajectory angles of needle direction for the new technique are consistent with those described by Hirahara et al. [18]. The presence of sex-related differences in needle trajectory angle in both the traditional and new techniques was expected as studies have often reported sex-related differences in pelvic and iliac crest anatomy [25-27]. Despite the presence of statistically significant sex-related differences, it is likely that these differences are not clinically significant as the trajectory angles differed by a small amount (within 1°-2.5° in all measurements).

Finally, we describe the area of bone marrow encountered by the needle trajectory, which averaged approximately 22.6 cm^2^. Due to the high volume of bone marrow required to obtain sufficient quantities of MSCs, aspiration is not only often conducted at the structures that have the most cancellous bone but also multiple aspiration attempts are frequently necessary. Therefore, if a second aspiration attempt is required while utilizing the new approach, we also describe the area of a second trajectory that is 30° cephalad to the trajectory (i.e., a second attempt angled away from the greater sciatic notch).

The strengths of our study included an objective and robust methodology by utilizing 3D reconstructed CT scans of the pelvis and sacrum to validate the safety of a recently described BMA technique. A comprehensive assessment of this approach was also described, including distance from the greater sciatic notch, degree of cephalad angulation and contralateral obliquity of the needle trajectory, area of bone encountered through needle trajectory, and area of bone encountered if a second-needle trajectory is required 30° cephalad for additional bone marrow.

However, this study also had several limitations. A retrospective study design was used and only Caucasian patients were included in our cohort. Although our initial sample size of 260 patients is reasonable, there was a considerable exclusion rate of 30%, due to unclear radiographic images or distorted anatomy, yielding a final sample of 182 patients. Inclusion of these patients with unclear radiographic imaging or distorted anatomy may have provided useful information on the actual variance, utilization, and safety of the new technique. Moreover, only the area for the new approach was measured and provided since the traditional approach frequently involves multiple aspiration attempts rendering a direct comparison as invalid. A volumetric measurement, instead of an area measurement, may be more useful in characterizing the needle trajectory but was not performed in this study. Finally, since we only utilized 3D reconstructed CT scans, this is a theoretical model study of a recently described BMA approach and warrants a true clinical study of outcomes, safety, and BMA quality in patients. Therefore, although the overall methodological quality and analysis of the retrospective study were high, these aforementioned shortcomings should be taken into consideration when interpreting findings from our study.

Future studies investigating this recently described and potentially useful technique are warranted, specifically well-designed randomized controlled trials that evaluate the efficacy and safety profile of BMA in patients.

## CONCLUSION

Compared to the traditional approach for BMA, a needle trajectory from the PSIS to the AIIS for BMA offers a wider safety margin from the greater sciatic notch if the needle trajectory enters the PSIS at approximately 20-30° cephalad from the transverse plane and 20-30° lateral from the midsagittal plane.

## Figures and Tables

**TABLE S1:**
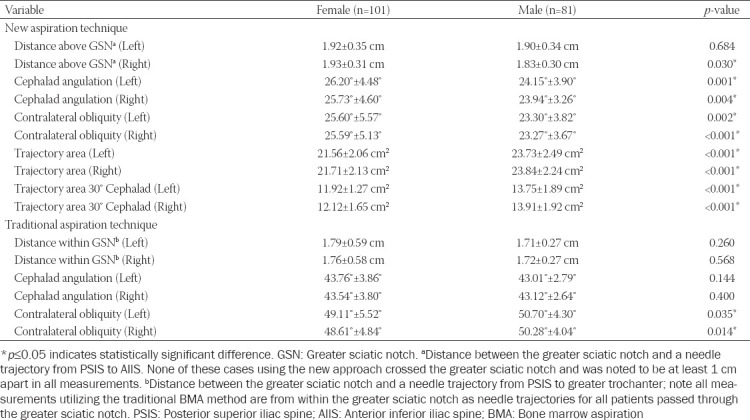
Gender-based comparisons of study measurements
